# Electrospinning preparation and electrical and biological properties of ferrocene/poly(vinylpyrrolidone) composite nanofibers

**DOI:** 10.3762/bjnano.4.19

**Published:** 2013-03-14

**Authors:** Ji-Hong Chai, Qing-Sheng Wu

**Affiliations:** 1 Department of Chemistry, Tongji University, Shanghai 200092, China; 2 Key Laboratory of Yangtze River Water Environment, Ministry of Education, Shanghai 200092, PR China; 3 Shanghai Key Laboratory of Molecular Catalysis and Innovative Materials, Fudan University, Shanghai 200433, PR China

**Keywords:** composites, electrochemistry, electrospinning, membranes, porous materials

## Abstract

Nanofibers containing ferrocene (Fc) have been prepared for the first time by electrospinning. In this paper, Fc was dispersed uniformly throughout the poly(vinypyrrolidone) (PVP) matrix for the purpose of combining the properties of PVP and Fc. The effects of solvents and Fc concentration on the morphologies and diameters of nanofibers were investigated. In the DMF/ethanol solvent, the morphologies of the obtained nanofibers significantly changed with the increase of Fc concentration. The results demonstrated that the morphologies of the nanofibers could be controlled through adjusting solvents and Fc concentration. Scanning electron microscopy (SEM) showed that the diameters of the obtained composite fibers were about 30–200 nm at different Fc concentrations. Thermogravimetric analysis (TGA) results confirmed the presence of ferrocene within the PVP nanofibers. X-ray diffraction (XRD) results showed that the crystalline structure of Fc in the fibers was amorphous after the electrospinning process. A biological evaluation of the antimicrobial activity of Fc/PVP nanofibers was carried out by using Gram-negative *Escherichia coli* (*E. coli*) as model organisms. The nanofibers fabricated by this method showed obvious antibacterial activity. Electrochemical properties were characterized based on cyclic voltammetry measurements. The CV results showed redox peaks corresponding to the Fc^+^/Fc couple, which suggested that Fc molecules encapsulated inside PVP nanofibers retian their electrochemical activity. The properties and facile preparation method make the Fc/PVP nanofibers promising for antibacterial and sensing applications.

## Introduction

Electrospinning is a simple and versatile technique for the production of polymers, composites and ceramic fibers [1–2]. Nanofibrous polymer materials produced by electrospinning have gained immense research interest because of their unique properties, such as high surface-area-to-volume and aspect ratios [3], and all kinds of electrospun fibers have already found a wide range of applications, including (but not limited to) sensors, catalysis, medicine and filtration [4–11].

Fc is a kind of organometallic compound with a “sandwich” structure [12], and it has attracted great interest from scientists because of its unique molecular structure and fascinating electronic properties and its potential applications in catalysis in organic synthesis, as functional materials, photosensitizers, stabilizers and conditioners, and in biochemistry and pharmaceuticals [13]. Fc decorated or incorporated into a polymer provides different properties compared with applying Fc alone. The incorporation of Fc in a polymeric matrix can improve the dispersion of Fc, increasing the catalyst effect and antibacterial activity of hybrid nanofibers. However, the current reports mainly focus on the preparation and application of various Fc-derivative nanofibers by the electrospinning technology [14–15]. To the best of our knowledge, Fc-PVP composites prepared by electrospinning have not been reported up to now. This may be due to the following two limiting factors: (a) most Fc derivatives are hydrophilic and easier to prepare by cheap, nontoxic polymers as its carrier compared to hydrophobic Fc; (b) in a single solvent, the solubility of the Fc is not good and the precursor solution is not easy to spin. PVP, one of the most utilized hydrophilic polymers, has a lot of important characteristics such as low toxicity, good compatibility, and excellent dissolvability in most organic solvents [16]. In this work, PVP polymer was selected as the carrier for immobilizing ferrocene. By incorporating Fc in PVP, the composite nanofibers would have enhanced wettability due to the presence of PVP. Additionally, the presence of well-dispersed Fc in PVP polymer is known to improve load transfer, thus the incorporation of Fc is expected to increase the catalytic properties of the electrospun Fc/PVP nanofibers. When an approach employing a conjugated solvent was simultaneously utilized, the Fc/PVP nanofibers were finally fabricated by electrospinning. The effects of Fc concentration and applied solvents on the size and morphology of the fibers were studied subsequently. Furthermore, some studies suggest that Fc possess antibacterial, electrochemical and catalytic properties so that the Fc/PVP nanofibers are likely to be useful in some applications such as antibacterial materials, sensors and catalysis.

## Results and Discussion

The processing parameters such as polymer concentration, Fc concentration, type of solvent, electrospinning voltage, polymer-solution flow rate, etc., have an important effect on the morphologies and structures of the fibers. By tuning the electrospinning parameters, fibers having a diameter from a nanometer to a micrometer in scale can be obtained, and the fibers can be facilely electrospun into different surface morphologies [17–19]. A preliminary investigation was carried out into electrospun PVP nanofibers. With a suitable concentration of PVP (about 2 to 25 wt %), fibers were obtained. On the other hand, when the concentration of PVP is below 2 wt %, “beads-on-strings” fibers are formed. With a high concentration of PVP (more than 25 wt %), the electrospinning was prohibitive such that no fibers were obtained. This was attributed to the high viscosity of the PVP solution [20]. In our experiments, 10 wt % PVP was chosen.

**Figure 1:**
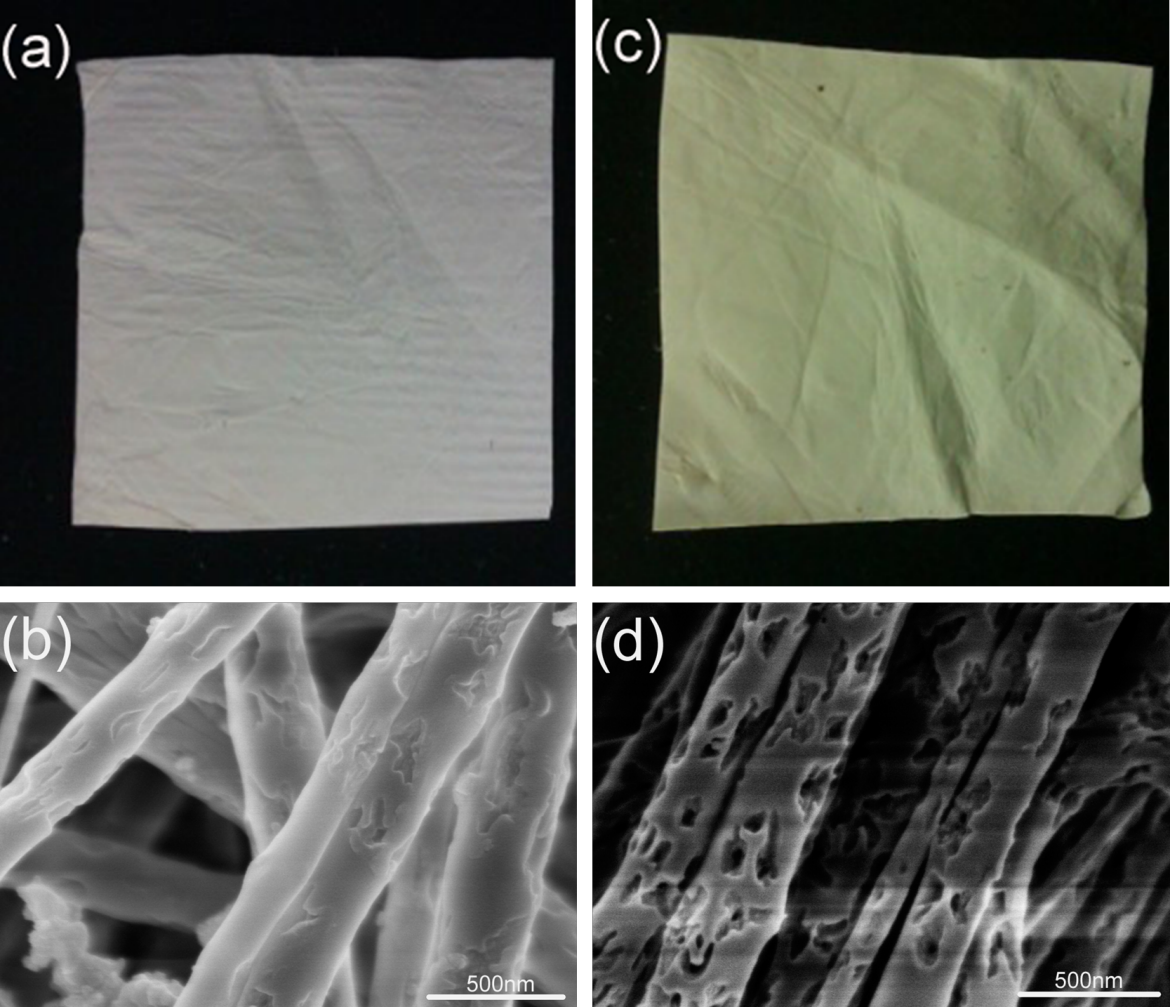
(a) An optical photograph of a blank PVP nanofiber membrane; (b) SEM image of the blank PVP nanofiber membrane; (c) an optical photograph of a composite Fc/PVP nanofiber membrane; (d) SEM image of the composite Fc/PVP nanofiber membrane. (voltage, 10 kV; work distance, 15 cm; flow rate, 1 mL/h).

Blank PVP nanofibers and composite Fc/PVP nanofibers were prepared by the electrospinning method. A nonwoven product consisting of PVP or Fc/PVP nanofibers was obtained. The nanofibers were processed into a thin membrane with an increase of the electrospinning time. Then the thin membrane obtained could be cut into any shape by a pair of scissors. The optical photographs and scanning electron microscopy (SEM) images of the products are shown in Figure 1. Figure 1a shows a blank PVP nanofiber membrane, and we could see that the membrane is white. Figure 1b reveals that these nanofibers interdigitate to form a feltlike film. The surface morphologies of fibers are rough and the diameter distribution is from 70 to 100 nm and tens of microns in length. Compared with the blank PVP nanofiber membrane, Figure 1c shows the optical photograph of the composite Fc/PVP nanofiber membrane. The Fc was loaded at an amount of 45 wt % with respect to PVP mass in the nanofibers, and the membrane clearly changed to pale yellow, which was attributed to the presence of Fc. The surface morphologies of the fibers became porous and the diameter obviously increased with the distribution ranging from 100 to 200 nm (Figure 1d). The high surface-to-volume ratio improved the antibacterial activity of the composite Fc/PVP nanofiber membrane.

**Figure 2:**
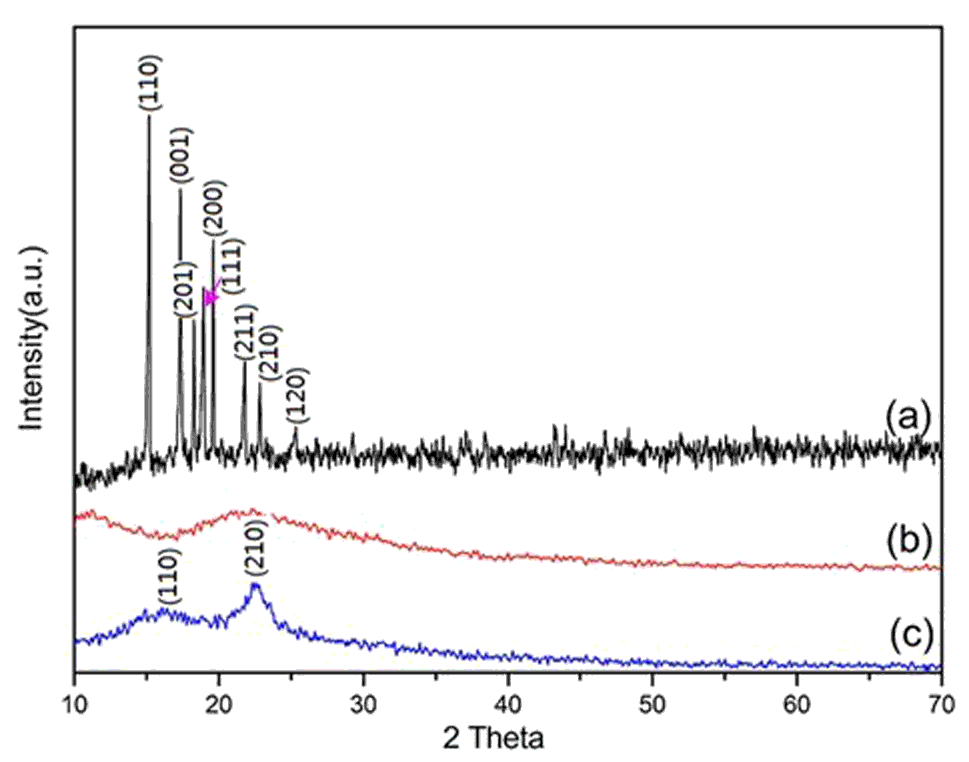
XRD pattern of (a) Fc powder; (b) blank PVP nanofibers; (c) Fc/PVP nanofibers.

As seen from the presented SEM micrographs (Figure 1d), no Fc crystals or aggregates of crystals were observed on the fiber surface. To demonstrate the physical state of Fc in the nanofibers, Fc powder, blank PVP nanofibers and Fc/PVP nanofibers were characterized by XRD. Figure 2 shows XRD patterns of the Fc powder, blank PVP nanofibers, and Fc-loaded PVP fibers. As shown in Figure 2a and Figure 2b, the Fc powder is crystalline with characteristic peaks at 2θ = 15.2, 17.3, 18.3, 18.9, 19.6, 21.8, 22.8 and 28°, and these are assigned to the corresponding (110), (001), (201), (111), (200), (211), (210) and (120) planes (ICDD PDF#29-1711), respectively. While blank PVP nanofibers are amorphous. Compared to the blank PVP nanofibers, there appeared two relatively weak and broad peaks at 2θ = 15.2° and 22.8° and no the other crystalline peaks of Fc appeared (Figure 2c), which shows that the amount of Fc encapsulated inside PVP nanofibers is very sufficient. However, the degree of crystallization of Fc in the composite fibers is not good. This is because the solidification of fibers is a very fast process with the volatilization of the solvents and the time is too short for Fc to fully crystallize during the electrospinning process. The results indicate that the Fc in the PVP polymer is in the amorphous form with a poor degree of crystallization.

**Figure 3:**
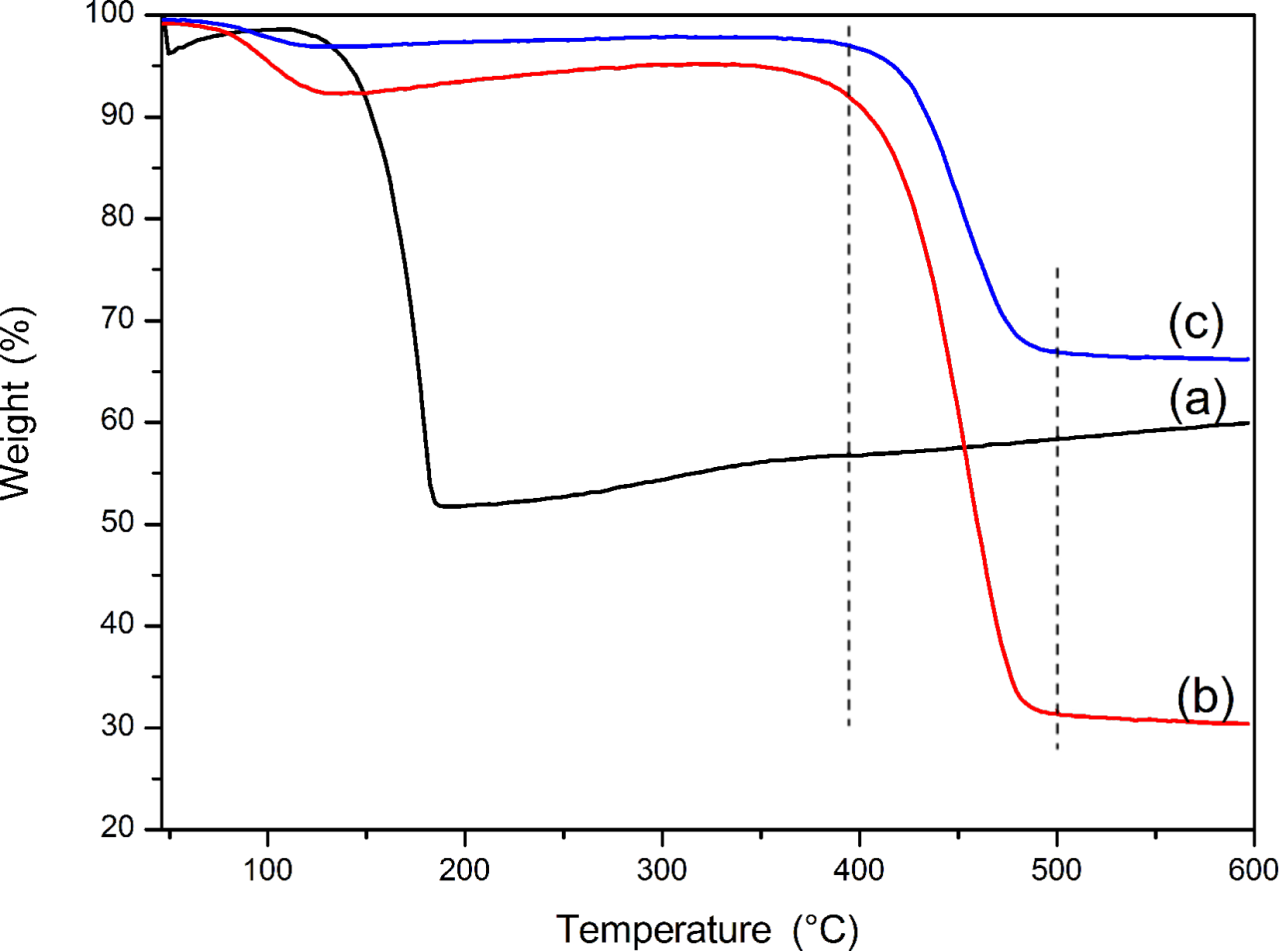
TGA thermograms of (a) Fc powder; (b) blank PVP nanofibers; (c) Fc/PVP fibers.

The degradation process of products was investigated using thermogravimetric analysis (TGA) from 20 to 600 °C in nitrogen gas. Figure 3a represents the TG curve of the pure Fc. It was found that the TGA data of Fc in nitrogen gas is different from the results obtained under air [21]. Weight loss (about 2%) before 100 °C is considered to be due to the evaporation of adsorbed water. The Fc is also part of the sublimation in the temperature range 100–200 °C due to the volatilizing of Fc above 100 °C [22]. However, Fc may start to form some kind of iron oxide/nitride and eventually be fixed after a certain time in nitrogen gas. The final weight loss is about 48 wt%. When the temperature further increases to 600 °C, the residue does not change. The blank PVP nanofibers show two significant steps of weight loss in the Figure 3b. The first weight loss is in the temperature range 20–100 °C, which is related to the release of solvents and moisture from the sample. The second weight loss starts at about 400 °C and finishes at almost 500 °C, which is caused by the decomposition of the PVP polymer [23]. Compared with the blank PVP nanofibers, a similar TG curve for Fc/PVP is observed (Figure 3c). The initial thermal decomposition temperature and the end temperature are consistent with those of the blank PVP fibers. However, the solid residue is much higher than that of the blank PVP fibers, which further confirms the presence of Fc within the PVP nanofibers.

In order to find the optimum preparation conditions for Fc-loaded PVP nanofibers, the influences of solvents and Fc loading on the morphologies and diameters of the electrospun fibers were investigated. The selection of a desirable solvent or solvent system is fundamental for the optimization of electrospinning. Different solvent systems are pivotal in determining the morphologies and diameters of the final fibers [24–26]. Various solvent systems were methodically developed.

**Figure 4:**
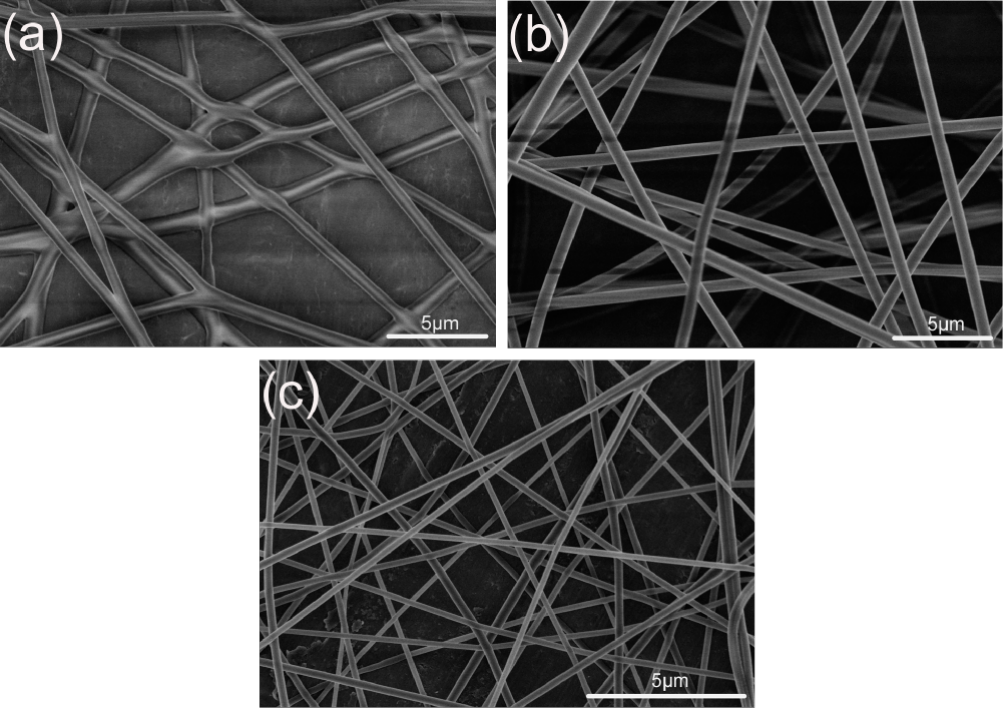
SEM images of Fc/PVP composite nanofibers from various solvent types of (a) pure ethanol; (b) ethanol/chloroform (v/v 1.3:1); (c) ethanol/DMF (v/v 11:9). The total PVP concentration is 10 wt % and the Fc concentration is 15 wt % with respect to PVP mass (voltage, 10 kV; work distance, 15 cm; flow rate, 1 mL/h).

Figure 4 shows the SEM images of the Fc/PVP nanofibers from ethanol, ethanol/DMF and ethanol/CH_2_Cl_2_ solvents. It shows that their size strongly depends on the type of solvents used. Junctions of nanofibers from ethanol are observed in the sample. The phenomenon can be well understood: wet nanofibers are not dried during the solidification process before they reach the collector, resulting in the junction morphology as shown in Figure 4a [27]. The surface of nanofibers is smooth with an average diameter of 500 nm. In contrast, the morphologies of nanofibers became more regular when different solvent systems were used. When CH_2_Cl_2_ was added to the solution, the fiber diameter increased significantly compared to the fiber produced from pure ethanol [28–29]. The average diameter was about 800 nm (Figure 4b). A narrow diameter distribution was observed. On the other hand, when CH_2_Cl_2_ was replaced by DMF, the fiber diameter decreased sharply to 170 nm with a smooth surface (Figure 4c). The diameters of fibers obtained from the three kinds of solvent systems are different, even though the electrospinning conditions (such as voltage, tip-to-collector distance and flow rate, etc.) and the concentration of solution are consistent. This is attributed to the boiling points and dielectric characteristics of the solvents.

It has been reported that a higher charge density can be induced on the jet surface by a larger solvent dielectric constant, which fully stretches the solution jet and yields more uniform and thinner nanofibers under the electrical field [30]. The boiling point of solvents has an important influence on the diameter of fibers. DMF has a high boiling point (BP 153 °C) and vapor pressure when compared to chloroform (BP 61.1 °C) and ethanol (BP 78 °C). In electrospinning, the lower the solvent boiling point is, the faster the evaporation rate of the solvent. Thus, it takes a shorter time for the jet to solidify, continuing the elongation of the jet. Hence, thick fibers deposited on the collector were commonly observed when highly volatile solvent systems such as ethanol/CH_2_Cl_2_ were used for the electrospinning. To obtain a smaller diameter of Fc/PVP nanofibers, we focused our attention on the ethanol/DMF solvent system.

**Figure 5:**
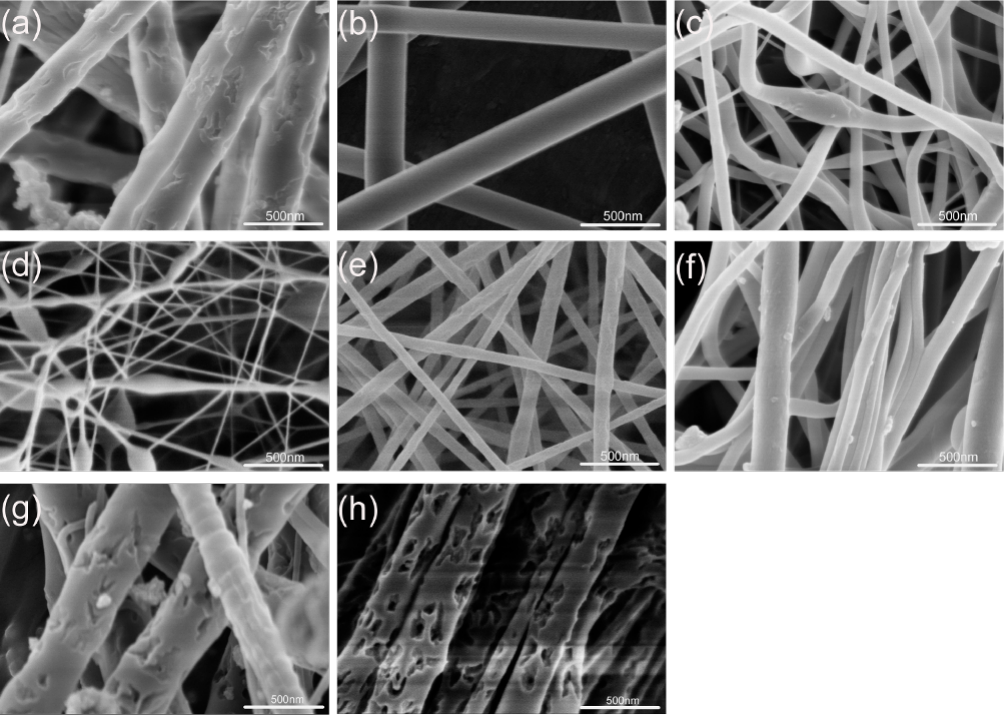
SEM images of the electrospun ferrocene/PVP fibers with different mass fractions of ferrocene: (a) 0 wt %; (b) 15 wt %, (c) 20 wt %, (d) 25 wt %, (e) 30 wt %, (f) 35 wt %, (g) 40 wt % and (h) 45 wt % (PVP concentration, 10 wt %; voltage, 10 kV; work distance, 15 cm; flow rate, 1 mL/h).

In the ethanol/DMF solvent system, significant morphological variations were observed for Fc/PVP nanofibers obtained from the same polymer concentration but having different Fc contents. Representative SEM images of electrospun Fc/PVP nanofibers are shown in Figure 5, illustrating the products fabricated from the different mass fractions of Fc. The morphologies and diameters of nanofibers are different for each sample, changing with the increasing content of Fc. Figure 5a–h shows an irregular trend in the change of the diameter of the fibers with the content of Fc increased from 0–45 wt %. Curved and rough morphologies are observed in the samples. However, irregular pores appeared on the surface of the fibers when the content of Fc was above 40 wt % (Figure 5g–h). The results show that the mass of Fc can clearly affect the surface morphologies of the fibers. It is considered that the viscosity and surface tension of the solution changes with increasing Fc content, which leads to this result [30–31]. The characterization of typical samples obtained was summarized in Table 1. In the experiment we found that when the amount of the Fc was more than 45% with respect to PVP, it was observed that Fc crystals appeared in the precursor solution. These crystals blocked the pinhole such that the electrospinning process was prohibited. We considered that the fibers possessed the biggest amount of Fc that can be loaded in the nanofibers. The sample containing 45 wt % Fc was selected as the final product for testing the electrochemical and antibacterial properties.

**Table 1:** Summary of surface morphology, structure and diameter of nanofibers by adjustment of the content of Fc.

Fc/PVP (w/w)	Morphology and structure of nanofibers	Fiber size (nm)

0.0 g/1.0 g	dense network structure; rough surface	70–100
0.15 g/1.0 g	dense network structure; very smooth surface	150–200
0.2 g/1.0 g	dense network structure; curved nanofibers; smooth surface	50–100
0.25 g/1.0 g	beads-on-strings structure; nanofibers with smooth surface	30
0.3 g/1.0 g	dense network structure; smooth surface	50–100
0.35 g/1.0 g	dense network structure; smooth surface	60–150
0.4 g/1.0 g	dense network structure; nanofibers with porous surface	100–200
0.45 g/1.0 g	dense network structure; nanofibers with irregular pores	100–200

**Figure 6:**
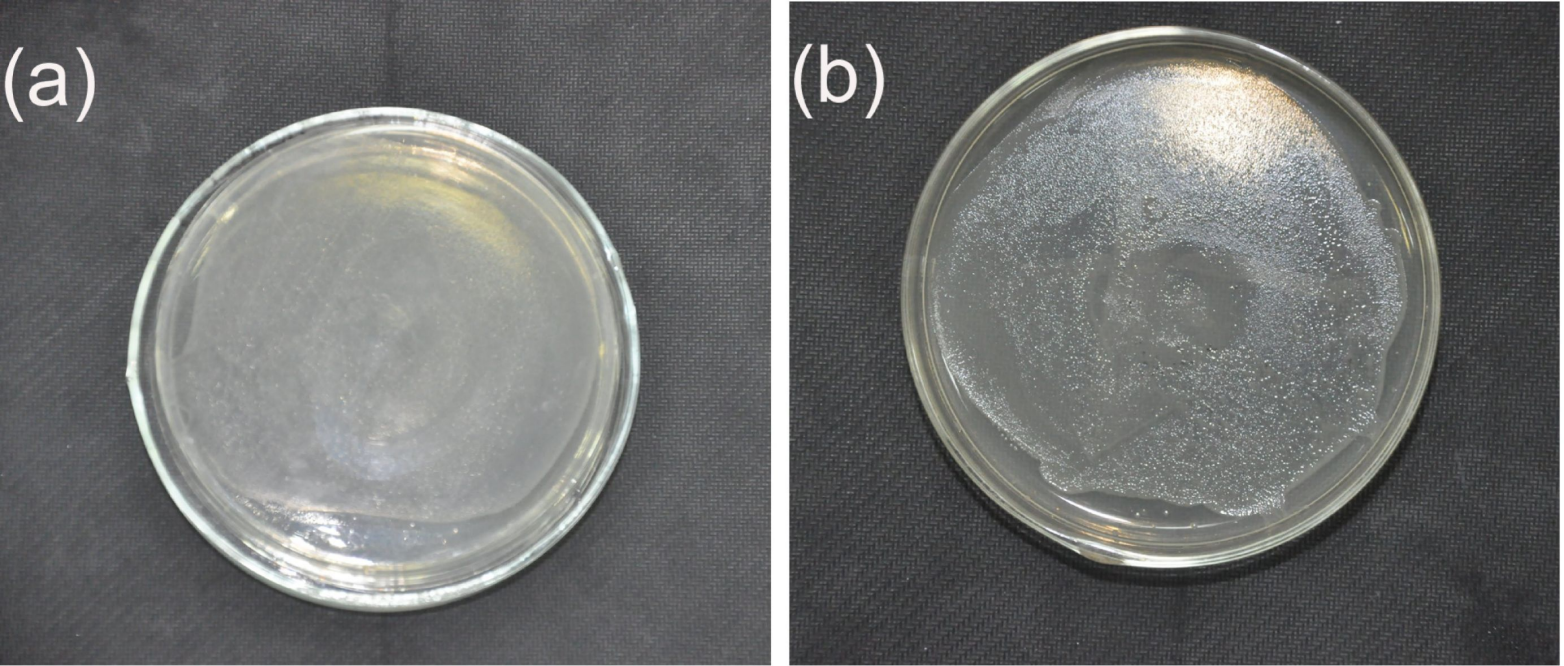
Photograph images of antibacterial tests against *E. coil* for (a) blank PVP nanofibers and (b) Fc/PVP nanofibers.

The Fc has antibacterial activity. But Fc molecules remain insoluble in aqueous medium and cannot diffuse through the medium, which limits the application of Fc as a bacteriostatic agent. In the case of Fc/PVP nanofibers, water-soluble polymer PVP, as a carrier, not only provides good dispersion for Fc, but also can release Fc quickly upon encountering a small amount of water. The antimicrobial activity of composite Fc/PVP nanofibers is explored in this article. Common *E.coli* was chosen for the experimental strains. Meanwhile we selected blank PVP nanofibers as the control experiment. The blank PVP nanofiber and composite Fc/PVP nanofiber webs were cut into discs with about 1 cm diameter and placed in the centre of the bacteria-inoculated agar plates. When in contact with the moist medium, the discs were dissolved due to the water-soluble PVP polymer. As can be seen from Figure 6a, the bacteria spread on agar plates where the blank PVP nanofiber disc was placed in the centre of the agar plate. Compared with blank PVP nanofibers, the composite Fc/PVP nanofibers placed in the centre of the agar plate killed the bacteria over and around them (Figure 6b), which showed that the composite Fc/PVP nanofibers obviously inhibited growth of the *E. coil*. It can be explained that Fc is lipophilic in nature and able to pass through the cell membrane. When *E. coli* is in contact with Fc, there are a series of chemical reactions between Fc and the enzymes, DNA and RNA in the cell due to the penetration of Fc, leading to cell death [32].

**Figure 7:**
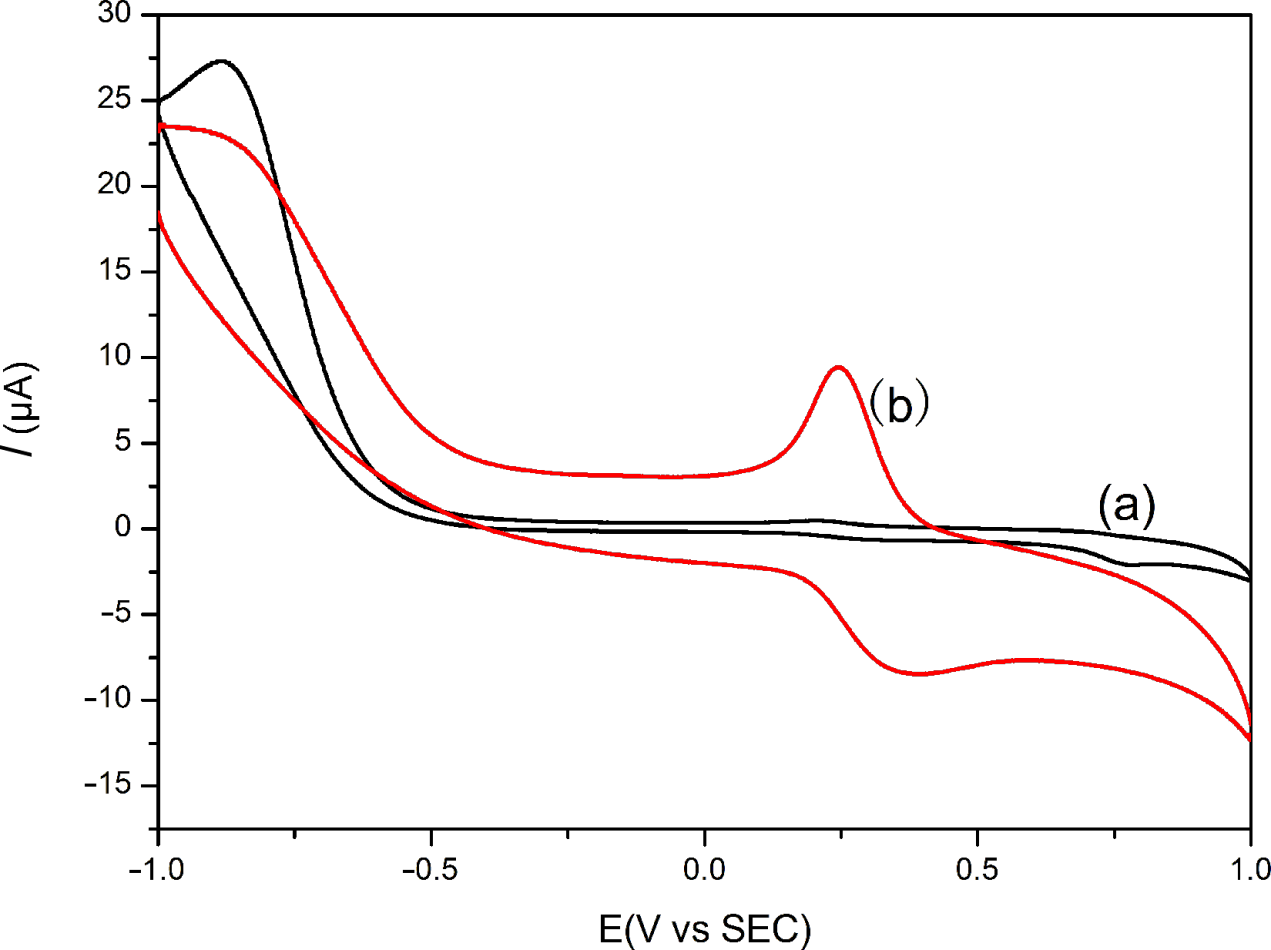
Cyclic voltammograms of (a) bare GC electrode and (b) modified electrodes with Fc/PVP nanofibers in tryptophan solution, *V* = 100 mV s^−1^.

**Figure 8:**
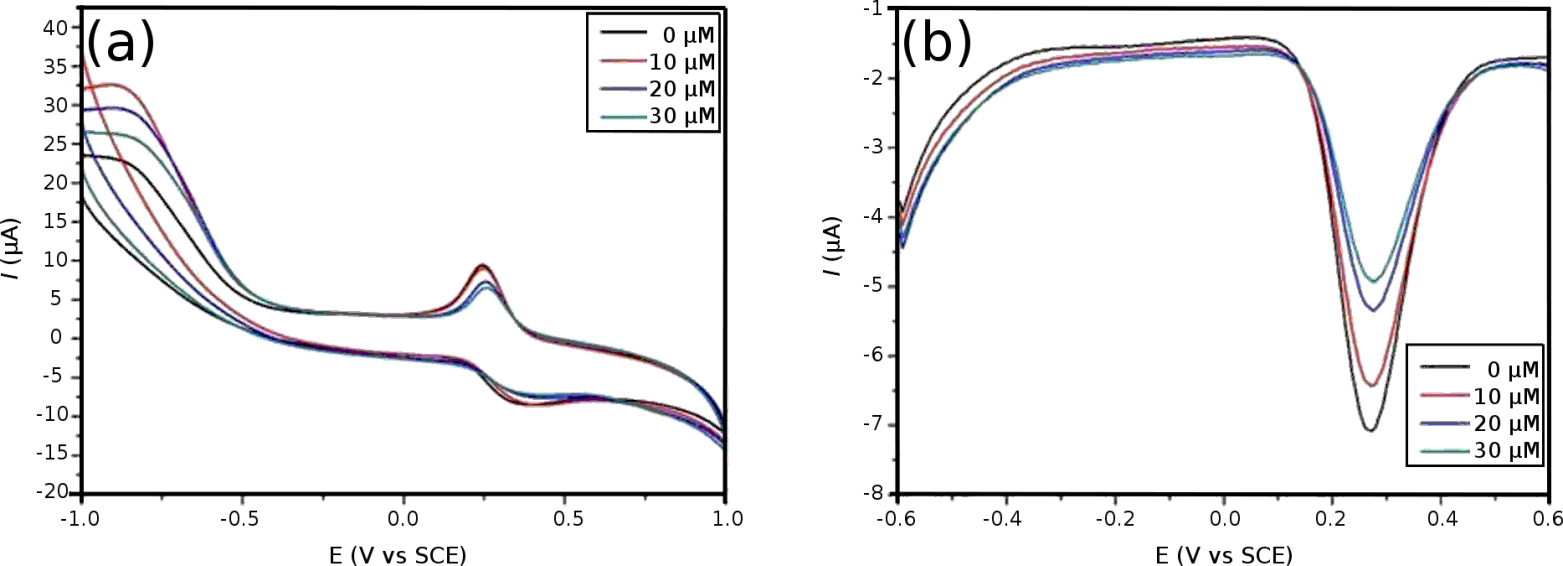
(a) Cyclic voltammograms for different concentrations of tryptophan in the Fc/PVP/GCE in PBS (pH 7), *V* = 100 mV s^−1^. (b) Cathodic differential pulse voltammograms for different concentrations of tryptophan at the Fc/PVP/GCE in PBS (pH 7), *V* = 100 mV s^−1^.

The Fc conveys many unique properties such as low toxicity, stability, lipophilicity, aromaticity and easy access to one-electron oxidation potential. Among these characteristics, the reversible redox property has a key application in the synthesis of several functionalized Fc derivatives having improved electrochemical, catalytic and sensing properties [33–35]. Figure 7 shows the cyclic voltammograms of Fc/PVP/GCE in PBS (pH 7) solutions. One pair of redox peaks of Fc with relatively good peak shape was observed (Figure 7b), which suggested the Fc/PVP/GCE acts as an electron-transfer mediator from the electrochemical characteristics [36]. Due to the small amounts of loaded ferrocene in the PVP nanofibers, the peak current is not high. Nevertheless, electrical communication was achieved between the electrode and the redox-active Fc molecules encapsulated inside the PVP nanofibers. It indicated that the molecular structure of Fc was not destroyed during the electrospinning process. In contrast, no redox peaks were observed on bare electrodes (Figure 7a) compared with modified electrodes. We further studied the electrochemical response of tryptophan on Fc/PVP/GCE in PBS (pH 7) solutions. CV responses for different concentrations of tryptophan in constant PBS (pH 7) solution are presented in Figure 8a. Results show that the anodic and cathodic peak currents have consistent reduction when increasing the tryptophan concentration. However, it is not easy to observe the diversifications due to very tiny current changes in the CV curves. Differential pulse-voltammetric experiments were performed to further support the above results (Figure 8b). The data show that there is also a gradual decrease in cathode current with an increase in tryptophan concentration. This could be explained by the fact that Fc^+^ has a strong coordinating capacity with tryptophan ions. It is relatively hard for the Fc^+^ to escape from the tryptophan ion, which ultimately affects the electrochemical behavior of redox centers [36]. This results in a decrease in current intensity.

## Conclusion

In summary, composite Fc/PVP nanofibers have been successfully prepared by electrospinning. The effects of the type of co-solvent and Fc concentration on the diameter and morphology of the fibers were studied. In the DMF/ethanol solvent, the diameter of the nanofibers was smaller. Increasing the content of Fc, the morphologies of the obtained nanofibers significantly changed, which was attributed to the variation of solution conductivity and solvent volatility. The results demonstrated that the morphologies and diameters of nanofibers could be controlled by adjusting the type of solvents and Fc concentration. These electrospun Fc/PVP nanofibers had bactericidal activity against the Gram-negative bacteria *E. coli*, and the glassy carbon electrode modified with Fc/PVP nanofibers displayed apparent electrochemical activity corresponding to the Fc^+^/Fc couple in PBS (pH 7) solution. When tryptophan was added, the peak current decreased with the increase of tryptophan concentration. The main reason was the coordination between Fc^+^ and tryptophan ion. Since the method demonstrated here is facile and a complex procedure is not involved in this work, the properties are not as good as reported for other materials. However, this work has provided us with a promising antibacterial and electrochemical sensing platform based on Fc/PVP composite nanofibers.

## Experimental

### Materials

Poly(vinylpyrrolidone) (*M*_w_ = 1,300,000) was purchased from Aldrich. Ferrocene was purchased from Shanghai Qingxi Chemistry and Technology Co., Ltd. (Shanghai, China). The solvents of *N*,*N*-dimethylformamide (DMF), dichloromethane (CH_2_Cl_2_) and ethanol (EtOH) were purchased from China Medicine (Group) Shanghai Chemical Reagent Corp (Shanghai, China). The above chemical reagents were analytical grade and used without further purification. Phosphate buffer solution (pH 7) was prepared by mixing Na_2_HPO_4_ and NaH_2_PO_4_ with distilled water. The strains used in this study were from our laboratory.

#### Preparation of nanofibers

Several spinning solutions were prepared by dissolving 1.0 g polymer and 0.15 g Fc in a single solvent or mixture of solvents, and the polymer concentration was fixed at 10 wt % in this paper. In the ethanol/DMF solvents, samples were prepared with Fc mass of 0, 0.15, 0.20, 0.25, 0.30, 0.35, 0.40 and 0.45 g in 10 wt % PVP solution. For each solution, they needed to be vigorous stirred for 6 h to a homogeneous orange solution. The obtained solution was then loaded into a plastic syringe equipped with a stainless steel needle with internal diameter of 0.6 mm. In the experiment, a voltage of 13 kV was applied for electrospinning. Aluminium foil served as the counter electrode with a tip-to-collector distance of 15 cm. The solution flow rate was 1 mL/h.

#### Characterization

SEM images were obtained on a Philips XL-30E scanning electron microscope (SEM) after gold sputter-coating. The XRD patterns of samples were determined with an X-ray diffractometer with Cu Kα radiation (*k* = 0.15405 nm, 40 kV, 100 mA) over the 2-theta angle range of 10–70 °C with a scanning rate of 10 °C/min. The TGA data was obtained by a DSC-STA 449C Jupiter thermal analysis instrument. About 4 mg sample in an aluminium pan was heated from 20 to 600 °C at a heating rate of 10 °C/min. All electrochemical measurements were performed with a CHI660A electrochemical workstation (Shanghai Chenhua Co., Ltd., China). A conventional three-electrode cell setup was employed with Fc/PVP/GCE as the working electrode, a platinum electrode as the counter electrode, and a saturated calomel electrode (SCE) as the reference electrode.

#### Antimicrobial activity testing of Fc/PVP nanofibers

The sample containing 45 wt % Fc was tested for antibacterial activity against the Gram-negative *E. coli*. Samples were prepared in the form of discs with a diameter of about 10 mm. Culture medium was prepared by mixing 3 g beef extract, 10 g peptone, 5 g NaCl, 16 g agar, and distilled water. After the activation, bacteria were inoculated and cultured in nutrient agar plate at 37 °C. For each bacterial suspension 100 µL was measured and evenly spread onto the solidified culture medium plate by the coating method. The Fc/PVP discs were placed onto the centre of substrates. The plates were then incubated at 37 °C for 24 h. Pictures of the plates were taken to illustrate the antibacterial performance of the samples.
